# New Details on Organophosphate Flame Retardants: Exposure in Men Appears Stable over Time

**DOI:** 10.1289/ehp.121-a168

**Published:** 2013-05-01

**Authors:** Kellyn S. Betts

**Affiliations:** Kellyn S. Betts writes about environmental contaminants, hazards, and technology for solving environmental problems for publications including *EHP* and *Environmental Science & Technology*.

A new study evaluating human exposure to two organophosphate flame retardants, tris(1,3-dichloro-2-propyl) phosphate (TDCPP) and triphenyl phosphate (TPP), suggests that metabolites from a single urine sample may be useful as biomarkers of longer-term exposure [*EHP* 121(5):580–585; http://dx.doi.org/10.1289/ehp.1205907]. Both TDCPP and TPP are widely used, and a limited number of animal studies suggest that both compounds may be carcinogenic, neurotoxic, or reproductive toxicants. Despite the potential for health effects, very few human studies of either flame retardant have been published to date, and the authors sought to characterize exposure over time to help inform future exposure assessments.

TDCPP and TPP have been used for decades, and their use is believed to have increased since polybrominated diphenyl ether (PBDE) flame retardants were banned in Europe and discontinued in the United States. Like PBDEs, TDCPP and TPP are not chemically bonded to the products they are intended to protect. This allows them to escape into indoor environments such as homes, offices, and car interiors. Indoor dust is known to be a significant source of exposure to PBDEs, and the new study suggests it may also be an important source of exposure to TDCPP, but not TPP. This may be because TPP has many other uses in addition to being a flame retardant in foam furniture padding.

The study’s 50 participants were from a larger project involving men recruited from a Boston infertility clinic. The men provided house dust samples, and 45 of them had archived urine samples that were analyzed for flame retardant metabolites. Previously, men from the same study who lived in homes with higher house dust content of TCDPP and TPP were shown to have reduced sperm counts and altered levels of hormones related to fertility and thyroid function.

**Figure:**
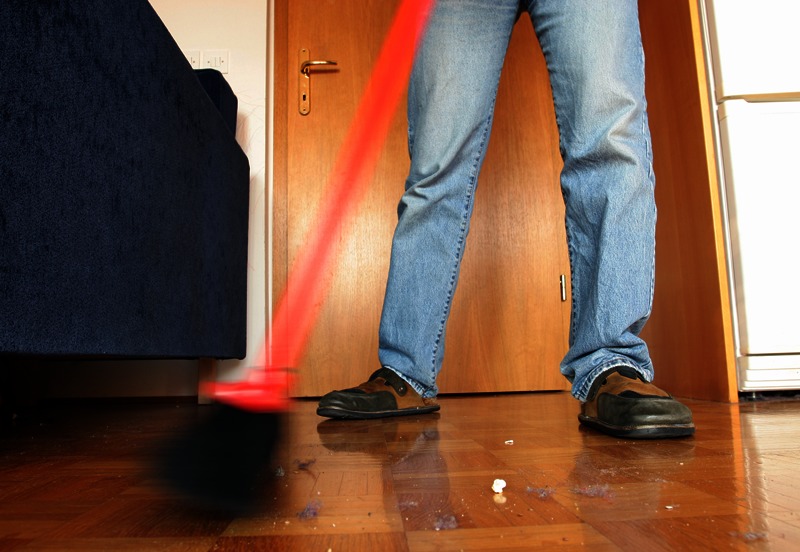
In a new study house dust appeared to be an important source of one organophosphate flame retardant but not the other—at least for men. © Tomaz Levstek/Thinkstock

The primary metabolite of TDCPP is bis(1,3-dichloro-2-propyl) phosphate (BDCPP), and the primary metabolite of TPP is diphenyl phosphate (DPP). Nearly all the house dust samples contained both TDCPP and TPP. The linked urine analysis found BDCPP in 91% of the samples and DPP in 96% of them. Although the highest concentrations of TPP in house dust exceeded the maximum level of TDCPP by 2 orders of magnitude, the pattern was reversed with their metabolites: The highest concentration of BDCPP in the urine samples was 3 times the maximum level of DPP.

The researchers also took advantage of a variability subset of the parent study in which men had submitted 9 urine samples over the course of 3 months. Analysis of the multiple samples for 7 men in the substudy indicated that urinary levels were generally stable over time. A single urine sample therefore may predict high exposure groups, although this needs to be confirmed in a larger sample.

The authors stress that there may be other sources of significant exposure to TDCPP outside the home, including vehicles and workplaces. They say that future studies should also investigate exposures among women and children, who may differ from men in exposure patterns and susceptibility.

